# The Guanine Exchange Factor *SsEFA6* Participates in Appressorium Formation and Virulence in *Sclerotinia sclerotiorum*

**DOI:** 10.3390/jof11110821

**Published:** 2025-11-20

**Authors:** Kunmei Wang, Ting Wang, Qi Xia, Na Xie, Jiancheng Cao, Shitou Xia

**Affiliations:** Hunan Provincial Key Laboratory of Phytohormones and Growth Development, College of Bioscience and Biotechnology, Hunan Agricultural University, Changsha 410128, China; yuhunan@stu.hunau.edu.cn (K.W.); tina@stu.hunau.edu.cn (T.W.); xiaqi@stu.hunau.edu.cn (Q.X.); naxie@stu.hunau.edu.cn (N.X.); caojiancheng@stu.hunau.edu.cn (J.C.)

**Keywords:** *SsEFA6*, mycelium, appressorium formation, virulence

## Abstract

*Sclerotinia sclerotiorum,* a soil-borne phytopathogenic fungus with a broad host range, often leads to severe disease and significant economic losses in agricultural production. The guanine exchange factor EFA6 of ADP-ribosylation factor 6 (ARF6) has been extensively studied in animals, but its function in fungi is seldom reported. Here, reverse genetics methods were employed to explore the effects of *SsEFA6* in the process of pathogenicity of *S. sclerotiorum*. Knockout of *SsEFA6* hindered appressoria formation and sclerotia production. However, it did not affect the secretion of oxalic acid, the sensitivity to cell wall inhibitors, or hyperosmotic stress. Nevertheless, *SsEFA6* deletion did result in a significant decrease in mutant virulence, indicative of its indispensability in virulence. Therefore, *SsEFA6* plays an essential role in appressoria formation, sclerotia production, and fungal virulence in *S. sclerotiorum*.

## 1. Introduction

*Sclerotinia sclerotiorum,* a notorious plant pathogenic fungus, poses a significant threat to agricultural production [1]. In the interaction between *S. sclerotiorum* and plants, a clear transition is observed from biotrophic to necrotrophic nutrition, spanning two distinct infection phases [2,3]. In the biotrophic infection phase, the transcription of genes such as *SsSte12* [4,5], *SsFkh1* [6], *SsTOR* [7], *SsAtg1* [8], and *SsNBR1* [9] in *S. sclerotiorum* prompts specific morphological changes at the tip of the mycelium, resulting in the formation of appressoria that penetrate the host’s epidermis. Concurrently, *S. sclerotiorum* secretes toxic factors, including oxalic acid [10], cell wall-degrading enzymes [11,12], and effectors [13,14,15,16], to impede the host’s pathogen recognition and defense mechanisms, thereby accomplishing colonization. Upon entering the necrotrophic nutrition phase, *S. sclerotiorum* releases a substantial quantity of ROS (reactive oxygen species) and virulence factors, which leads to prompt death of host cells and the spread of necrotic lesions [2,3,17,18]. Although considerable progress has been achieved in understanding the pathogenic mechanisms of *S. sclerotiorum*, many mechanisms are still to be uncovered.

The guanine exchange factor of ADP-ribosylation factor 6 (EFA6), encompassing Sec7 and pleckstrin domains, regulates endomembrane cycling and promotes the redistribution of transferrin receptors on the cell surface in animal systems [19]. In polarized epithelial cells, EFA6 is closely associated with the formation of tight junctions (TJ), and its accumulation increases in line with the speed of TJ formation [20]. Moreover, studies suggest that EFA6 regulates the activation of Arf6 and clathrin-mediated endocytosis by recruiting cytosolic proteins to flat regions of the plasma membrane [21]. Recent findings indicate that EFA6 participates in the early stages of cilium formation by promoting the formation of distal appendage vesicles on ciliary vesicles.

However, in the plant pathogenic fungus *S. sclerotiorum*, the role *SsEFA6* plays is hardly known [22]. Xu et al. developed a pipeline strategy combining forward genetic screening and high-throughput NGS (next-generation sequencing), which showed outstanding performance in the discovery of pathogenic genes in the *S. sclerotiorum* [4,23,24,25]. Utilizing this method, a weak mutant *2WX2-11* of *S. sclerotiorum* was screened and identified with 3 candidate genes. Among these, one candidate gene, encoding a protein containing the Sec7 and PH_9 domains, caught our attention. Proteins containing SEC7 domains are annotated as guanine nucleotide exchange factors (GEFs) specific to ADP-ribosylation factors (ARF), which are crucial for vesicular protein trafficking. Growing evidence suggests a close relationship between vesicle transport pathways and the virulence of plant pathogenic fungi [26,27,28,29,30]. Hence, in this study, we utilized reverse genetics to characterize *SsEFA6* in *S. sclerotiorum.* Evolutionary analysis of the *Ss*EFA6 protein indicates its conservation in plant pathogenic fungi *Botrytis_cinerea*, *Monilinia_fructicola*, and *Monilinia_laxa*. Knockout of *SsEFA6* did not impair hyphal growth significantly, but increased the formation of branches and aerial hyphae. Surprisingly, *SsEFA6* knockout also inhibited the formation of sclerotia and appressoria but did not alter *S. sclerotiorum*’s sensitivity to osmotic stress. Most importantly, the knockout mutant exhibits significantly reduced virulence on the hosts.

## 2. Materials and Methods

### 2.1. Fungal Strains and Growth Conditions

*S. sclerotiorum* 1980 (WT), *SsEFA6-C* (complementary strain), and *ΔSsefa6* strains were cultivated on potato dextrose agar plates (potato powder 5 g/L, glucose 20 g/L, agar 15 g/L, and chloramphenicol 0.1 g/L, Bio-Way Technology, Shanghai, China) and incubated at a constant temperature of 20 °C. To store *SsEFA6-C* and *ΔSsefa6* strains at 4 °C for an extended period, PDA plates were supplemented with 200 µg/mL hygromycin B (Roche, Basel, Switzerland) and 100 µg/mL G418 sulfate (Geneticin, Yeasen, Shanghai, China).

### 2.2. Plant Resources and Cultivation Conditions

Seedlings of *Arabidopsis thaliana* (Col-0) and *Nicotiana benthamiana* were grown in a growth chamber set to 22 °C, with a light cycle of 16 h followed by 8 h of darkness. Following a four-week growth period, the plants were employed for virulence testing with *S. sclerotiorum*.

### 2.3. Screening of Infection-Deficient Mutants

Referring to the methodology proposed by Xu et al. [23], this study involved the collection of ascospores from the wild-type strain *S. sclerotiorum* 1980. The ascospores were diluted to a concentration of 10^4^ spores/mL using sterile PDB medium and evenly spread on PDA plates. Subsequently, the plates were exposed to a UV dose of 9000 mJ/cm^2^ for 15 s and incubated at 20 °C for 2 to 3 days, following which individual colonies were selected and transferred to a 96-well plate. After 7 days of cultivation, mature sclerotia were harvested and stored in labeled storage bags. Prior to the screening process, the sclerotia sterilized (using disinfectant solution, 15% sodium hypochlorite + 1‰ Tween 20) were stored in the dark at 4 °C for 3 to 4 days and then transferred to PDA plates for further cultivation. Upon germination of young hyphae, preliminary screening was conducted by inoculation on *Lactuca sativa* var. *asparagina* L.H.Bailey ex Holub to select a population of strains with significantly reduced infectivity to the host. Multiple repeated experiments were conducted on *L. sativa* and *N. benthamiana* to confirm the stable reduction in infectivity in the potential mutant strains.

### 2.4. Sequencing of the Mutant Genotype Genome

Fresh fungal mycelia of *S. sclerotiorum* mutant strains with significantly reduced infectivity were inoculated into potato dextrose broth (PDB) and cultured in a constant temperature incubator at 20 °C for 5 to 6 days. Subsequently, the mycelia floating on the surface of the culture medium were collected and ground into fine powder in liquid nitrogen. Genomic DNA of the mutant and wild-type strains was extracted using the CTAB (cetyltrimethylammonium bromide) approach [24]. The crude DNA samples were further purified by commercial services (Novogene, Beijing, China) to meet the requirements of next-generation sequencing (NGS). The degradation and contamination of DNA were evaluated using a 1% agarose gel. Then, the paired-end sequencing libraries were constructed and sequenced by Novogene using the Illumina NovaSeq 6000 sequencer (Illumina, San Diego, CA, USA). The obtained clean reads were aligned to the reference genome of the nuclear disk fungus (ASM185786v1).

### 2.5. Identification of Candidate Genes

Sequence reads obtained using next-generation sequencing technology were aligned to the reference genome of the *S. sclerotiorum* 1980 strain. Mutant sites were identified using SAMtools software (1.12) (default settings) [31]. Lineage-specific short variant detection (SNP and INDEL) was performed following the GATK pipeline [32] and annotated. Following the method described by Li et al. [24], false positive mutations in repetitive sequences were excluded.

### 2.6. Validation of Candidate Gene Mutation Sites Through Sanger Sequencing

Genomic DNA of strain 2WX2-11 was extracted using the CTAB method and used as a template for amplification of the gene fragment *sscle_12g090120* using SeqF_120, SeqF_360, SeqF_780, SeqR_120, SeqR_360, and SeqR_780 primers (Table S1). Subsequently, PCR products were sent to Sangon Biotech for Sanger sequencing using universal primers.

### 2.7. Bioinformatics Investigation of SsEFA6

The protein APA14242 encoded by the gene *sscle_12g090120* was analyzed for conserved domains using the interpro/database (https://www.ebi.ac.uk/, Accessed on 10 April 2025). The BLASTP (2.12.0) [33] tool was utilized to identify homologous proteins of APA14242 in *S. sclerotiorum*, and MEME 5.5.1 [34] software was employed to recognize conserved motifs within the protein sequence of APA14242. A phylogenetic tree was constructed using the Neighbor-Joining method in MEGA 11 [35] and visualized using iTOL V6 [36] software. Additionally, the Python (3.12) version of the MCScan (JCVI toolkit) tool [37] was used for collinearity analysis and visualization of the proteins APA14242, APA13170, APA12987, and APA15292 in the plant pathogenic fungi *B. cinerea*, *Fusarium graminearum*, *Fusarium oxysporum*, *Magnaporthe oryzae*, *Monilinia fructicola*, and *S. sclerotiorum*. The interaction network and enrichment analysis of *Ss*EFA6 protein were conducted using STRING (https://cn.string-db.org, accessed on 20 May 2025). The expression profile of sscle_12g090120 (*SsEFA6*) was investigated utilizing the dataset GSE159792 [38] from the GEO database (https://www.ncbi.nlm.nih.gov/geo/, accessed on 20 May 2025).

### 2.8. Knockout and Complementation of SsEFA6

The split-marker strategy was applied to remove the *SsEFA6* gene from *S. sclerotiorum*. As outlined earlier [39], the replacement fragments *SsEFA6*-UP-HY and *SsEFA6*-Down-YG were constructed using two rounds of PCR to substitute the target gene, SsEFA6. Following this, the replacement fragments were co-transformed into protoplasts of the WT strain. To obtain knockout mutant homozygotes, *SsEFA6* knockout transformants were selected on PDA plates with 200 µg/mL hygromycin B, and underwent at least three rounds of hyphal tip transfer and protoplast purification.

For genetic complementation, the homologous recombination primers EFA6F and EFA6R were designed using Snap Gene software (6.11) to include 20 bp sequences adjacent to the *Xho*I and *Kpn*I restriction enzyme sites on the vector PCH-EF-1-Neo (modified from PCH-EF-1 provided by D. Jiang from Huazhong Agricultural University). The full-length sequence of *SsEFA6* and the 2 kb sequence upstream of the promoter were amplified by PCR. The amplified fragment was ligated into the vector PCH-EF-1-Neo using the homologous recombination enzyme Exnase II. The recombinant plasmid was co-transformed into protoplasts of the knockout mutant strain. Six sets of primers (Table S1) were used in PCR for validation, and the transformed knockout and complementary strains were chosen for subsequent experiments.

### 2.9. DNA and RNA Manipulation

*S. sclerotiorum* mycelium was cultured on PDA medium with cellophane for 48 h, then ground into powder using liquid nitrogen. Genomic DNA from wild-type (WT) and mutant strains was extracted via the cetyltrimethylammonium bromide method [23]. The wild-type genomic DNA extracted was utilized as a template for amplifying both the full-length sequence and the flanking sequence of *SsEFA6*. Genomic DNA extracted from the mutants served to verify the knockout and complementation of *SsEFA6*. To assess the transcriptional expression of *SsEFA6* in the knockout and complementation strains, mycelium was harvested following 48 h of cultivation on PDA medium overlaid with cellophane. Total RNA was subsequently extracted using the Steady Pure Plant RNA Extraction Kit (Accurate Biology, Changsha, China). First-strand complementary DNA (cDNA) was synthesized from the total RNA using the Evo M-MLV RT-PCR Kit (Accurate Biology, Changsha, China) as the template. Semi-quantitative reverse transcription PCR (RT-PCR) was conducted using cDNA as the template for 28 PCR cycles. All primers employed in this study are detailed in Table S1.

### 2.10. Observation of Colony Morphology

Mycelial plugs (5 mm) of WT, *∆Ssefa6*, and *SsEFA6-C* were placed in the center of 9 cm PDA Petri dishes and incubated at 20 °C. Mycelial growth was measured every 24 h over 48 h, and the average growth rate was calculated. Colony and sclerotia morphologies were photographed at 2, 4, 6, 8, and 15 days. The experiment was repeated three times with three technical replicates each.

### 2.11. Stress Treatment

Mycelial plugs (5 mm) from WT, ∆Ssefa6, and SsEFA6-C strains were placed in the center of 9 cm Petri dishes with PDA medium containing Congo Red and osmotic stressors (0.5 M NaCl, KCl, 1 M sorbitol, and glucose). The plates were incubated at 20 °C. After 48 h, the mycelium growth radius was measured, and the inhibition rate was calculated as 100 × (colony radius on pure PDA—colony radius under stress)/colony radius on pure PDA. Colony morphology was photographed, and the experiment was independently repeated three times with three technical replicates each.

### 2.12. Evaluation of Compound Appressoria, Oxalic Acid Secretion, and Host Plant Pathogenicity

Mycelial plugs (5 mm) from WT, *∆Ssefa6*, and *SsEFA6-C* strains were placed on PDA medium with 0.005% (*w*/*v*) Bromo-phenol Blue. After incubating at 20 °C for 48 and 72 h, color changes were photographed, with three replicates.

To evaluate appressorium development, 8 mm mycelial plugs from WT, *∆Ssefa6*, and *SsEFA6-C* strains were placed on slides and transferred to 9 cm Petri dishes containing a moistened paper towel with 11 mL sterile water. These dishes were incubated at 20 °C. After 24 h, appressoria morphology was examined with an optical microscope. After 48 h, appressoria were photographed using a digital camera, with three replicates.

To study the structure of compound appressoria on onion epidermal cells, 5 mm mycelial plugs from WT, *∆Ssefa6*, and *SsEFA6-C* strains were inoculated on onion epidermis in 9 cm Petri dishes with a moistened paper towel. Cultures were incubated at 20 °C for 16 h, then stained with 0.5% Trypan Blue for 30 min and decolorized with a 3:1:1 ethanol, acetic acid, and glycerol solution. As previously outlined, the morphology of the compound appressoria was analyzed under an optical microscope [40]. The experiment was conducted independently three times.

For the virulence analysis, 2 mm and 5 mm mycelial plugs were taken from WT, *∆Ssefa6*, and *SsEFA6-C* strains, respectively. The 2 mm plugs were used to inoculate detached *A. thaliana* leaves, while the 5 mm plugs were used for wounded *N. benthamiana* leaves. The latter were placed in 9 cm square Petri dishes with a 25 cm moistened paper towel saturated with 11 mL of sterile water. In a 22 °C growth room with a 16 h light and 8 h dark cycle, infection morphology was photographed at 36- and 48 h post-infection. Lesion areas were analyzed using ImageJ 1.46r [41] to determine area reduction. The experiment was independently repeated at least two times, each with three technical replicates.

## 3. Results

### 3.1. Identification of Candidate Genes Responsible for the Phenotype of the 2WX2-11 Mutant

Following UV-mutagenesis of haploid ascospores of *S. sclerotiorum*, a population of mutant strains was generated. Subsequent screening on *L. sativa* led to the identification of a mutant strain with reduced virulence, designated as 2WX2-11 (Supplementary Figure S1A). Subsequent multiple rounds of virulence testing on *L. sativa* and *N. benthamiana* revealed a significant decrease in virulence of the mutant compared to the wild type strain, with stable phenotypes (Supplementary Figure S1B). To pinpoint the mutation sites responsible for the phenotype of the 2WX2-11 mutant, whole-genome sequencing (NGS) analysis was conducted. Through genome-wide sequence alignment and manual screening to exclude synonymous mutations, intronic mutations, intergenic mutations, and non-homozygous mutations, only three exonic SNPs remained in the 2WX2-11 genome, all resulting in non-synonymous amino acid changes. These three SNPs located in *sscle_01g009360* (S67L, serine to leucine mutation at position 67), *sscle_04g033780* (D588Y, aspartic acid to tyrosine mutation at position 588), and *sscle_12g090120* (S1104F, serine to phenylalanine mutation at position 1104), respectively, were confirmed as the candidate mutation sites for further reverse genetics verification, by Sanger sequencing of these three SNPs (Supplementary Figure S1C,D).

### 3.2. SsEFA6 Differs Evolutionarily Among Plant Pathogenic Fungi and Expresses Differently During the Infection Process

**Figure 1:**
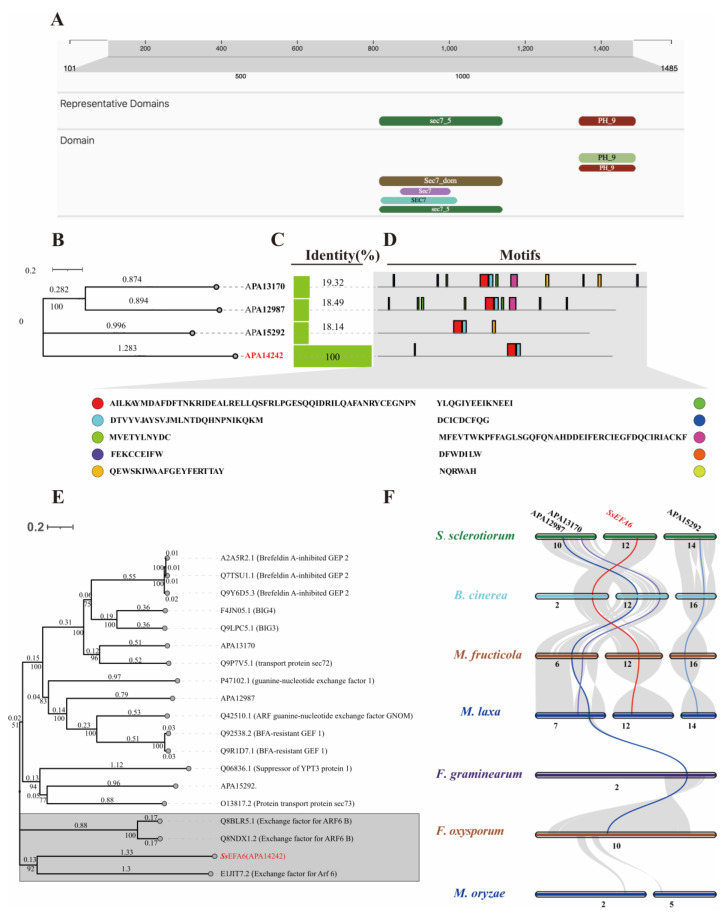
The bioinformatics analysis of *SsEFA6.* (**A**) Conservative domain analysis. (**B**) Identification of SEC7 domain proteins in *S. sclerotiorum*. (**C**) Homology analysis of SEC7 domain proteins in *S. sclerotiorum*. (**D**) The conservation motif analysis of SEC7 domain proteins in the *S. sclerotiorum*. (**E**) Phylogenetic tree analysis of SEC7 domain proteins across multiple species. (**F**) Genomic collinearity analysis of different plant pathogenic fungi. Chromosome numbers are labeled at the bottom of each chromosome. The colored bars represent chromosomes of different species. The gray lines in the background indicate collinear blocks between genomes of two species connected by gray lines, while the colored lines highlight homologous gene pairs encoding Sec7 domain proteins.

As one of the candidate genes of the weak mutant 2WX2-11 in *S. sclerotiorum* (Supplementary Figure S1), *sscle_12g090120* was found to encode a protein containing the Sec7 and PH_9 domains (Figure 1A), which were annotated as guanine nucleotide exchange factors (GEFs) specific to ADP-ribosylation factors (ARF). Analysis of the protein (APA14242) encoded by *sscle_12g090120* in *S. sclerotiorum* revealed the presence of 3 homologous proteins, namely APA13170, APA12987, APA15292 (Figure 1B,C). Additionally, it was observed that the protein APA14242 contains fewer motifs compared to the 3 homologous proteins (Figure 1D). Phylogenetic analysis of SEC7 domain-containing proteins in *S. sclerotiorum* and known functional Sec7 domain proteins from various species, using the maximum likelihood method, showed that the protein APA14242 was closely related to and clustered together with the exchange factor for ADP-ribosylation factor 6 (Arf6) (Figure 1E). Hence, the protein APA14242 encoding gene *sscle_12g090120* was named *SsEFA6*. Furthermore, it was found that *Ss*EFA6 and its homologous proteins exhibited close relationships with SEC7 domain-containing proteins in plant pathogenic fungi *B. cinerea*, *M. fructicola*, and *M. laxa*, being evolutionarily conserved, but showing a distant relationship from *F. graminearum*, *F. oxysporum* and *M. oryzae,* indicating some evolutionary loss (Figure 1F).

**Figure 2:**
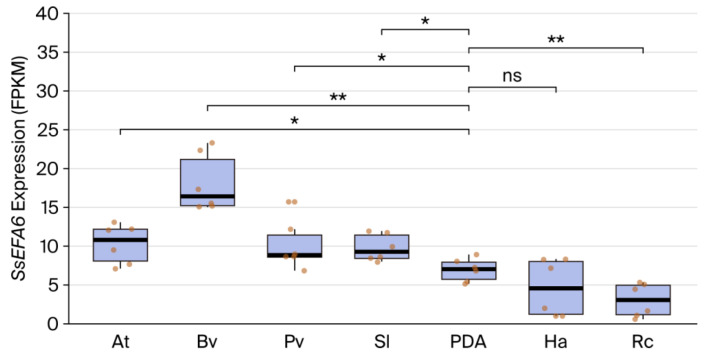
Expression pattern analysis of *SsEFA6*. *At*, represents *A*. *thaliana*; *Bv*, *Beta vulgaris* (beetroot); *Pv*, *Phaseolus vulgaris* (Common bean); *Sl*, *Solanum lycopersicum* (tomato); PDA, Potato Dextrose Agar Medium; *Ha*, *Helianthus annuus* (sunflower); and *Rc*, *Ricinus communis* (castor bean). The data were obtained from the GEO database with the accession number GSE159792. Statistical significance between PDA and plants was evaluated utilizing One-way ANOVA (n = 6, * *p* < 0.05, ** *p* < 0.01, ns, not significant).

Based on the transcriptome FPKM (fragments per kilobase of transcript per million mapped reads) expression data of *S. sclerotiorum* and plant interaction process, the expression pattern of SsEFA6 was investigated, and the results revealed that, compared to pure PDA, *SsEFA6* shows significantly increased expression levels in the interactions with *A. thaliana*, *Beta vulgaris*, *Phaseolus vulgaris*, and *Solanum lycopersicum*, while it is significantly downregulated in *Ricinus communis* (Figure 2). This suggests that *SsEFA6* plays a positive role in the interaction process between *S. sclerotiorum* and plants.

### 3.3. SsEFA6 Plays a Role in the Formation Process of Sclerotia

**Figure 3:**
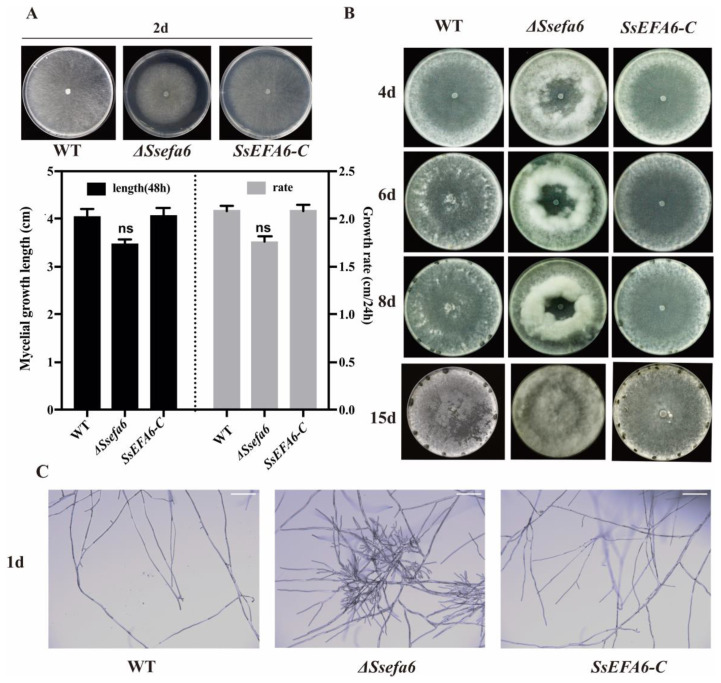
Phenotypic analysis of the *S. sclerotiorum.* (**A**) Determination of mycelial growth rate. (**B**) Observation of colony morphology. (**C**) Observation of mycelial branching pattern. Scale bar, 10 mm. Error bars show the standard deviation. The experiment was repeated at least three times, with results that were consistent. Statistical analysis comparing the wild-type (WT) and knockout mutant strains or complementary strains was carried out using Student’s *t*-test (*p* < 0.05, ns, not significant). WT represents the wild-type strain, *ΔSsefa6* denotes the knockout strain, and *SsEFA-C* represents the complemented strain.

To elucidate the role of the *SsEFA6* in *S. sclerotiorum,* we generated the knockout mutant *ΔSsefa6* and then the complemented mutant *SsEFA6-C* through homologous recombination-based split-marker (Supplementary Figures S2 and S3) and transgenic complementation approaches (Supplementary Figure S4), respectively. Phenotypic analysis of the *ΔSsefa6* strain revealed a slightly slower growth rate of mycelia, compared to the wild type (WT) and *SsEFA6-C* strains (Figure 3A), but the difference was not significant. However, deletion of the *SsEFA6* gene was found to impair the formation of sclerotia in *S. sclerotiorum*. While sclerotia formation could be clearly observed in the WT and *SsEFA6-C* strains on potato dextrose agar (PDA) medium, with mature sclerotia visible by days 8 and 15, the *ΔSsefa6* strain only exhibited abundant aerial hyphae, and no sclerotia formation was observed (Figure 3B). Additionally, the *ΔSsefa6* strain formed more numerous shorter branches, compared to WT and *SsEFA6-C* strains (Figure 3C). These findings suggest that *SsEFA6* is involved in the process of sclerotia formation.

### 3.4. SsEFA6 Is Essential for Compound Appressorium Formation but Dispensable for Oxalate Secretion

**Figure 4:**
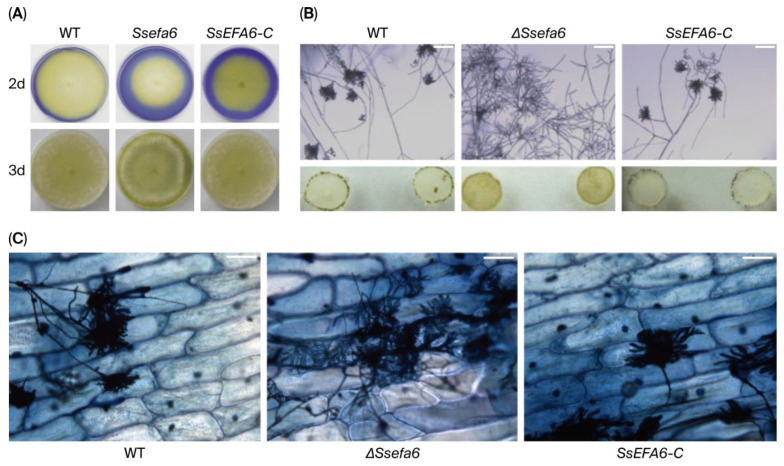
Oxalate secretion and appressorium formation analysis. (**A**) Evaluation of oxalate acid production ability. Strains *ΔSsefa6*, WT, and *SsEFA6-C* were inoculated and cultured on PDA-BPB plates (containing 0.005% (*w*/*v*) Bromophenol Blue), and the color changes in the PDA-BPB plates were observed after 2d and 3d of cultivation (bromophenol blue turns yellow at pH 3.0–4.6 and blue at pH > 4.6). (**B**,**C**) Analysis of infection structures. Observations of the compound appressorium around the fungal blocks on cover slips, 24 h and 48 h after inoculation with WT, *ΔSsefa6*, and *SsEFA6-C* strains; microscopic observation of the compound appressorium on onion epidermal cells 24h after inoculation. Scale bar, 10 mm. The experiment was replicated at least three times, yielding consistent results. Error bars indicate standard deviation. WT denotes the wild-type strain, *ΔSsefa6* refers to the knockout strain, and *SsEFA-C* represents the complemented strain.

To further test whether the deletion of the *SsEFA6* affects appressorium formation and oxalate secretion, their oxalate production, appressoria development, and penetration ability were examined. As shown in Figure 4A, no significant difference was found among *ΔSsefa6*, WT, and *SsEFA6-C* strains, indicating that *SsEFA6* is dispensable for oxalate secretion in *S. sclerotiorum*. However, the formation of normal appressoria was not observed in the *ΔSsefa6* strain, compared to WT and *SsEFA6-C* strains (Figure 4B,C), demonstrating an essential role of *SsEFA6* in appressorium formation of this fungus.

### 3.5. SsEFA6 Participates in the Pathogenic Process of S. sclerotiorum

**Figure 5:**
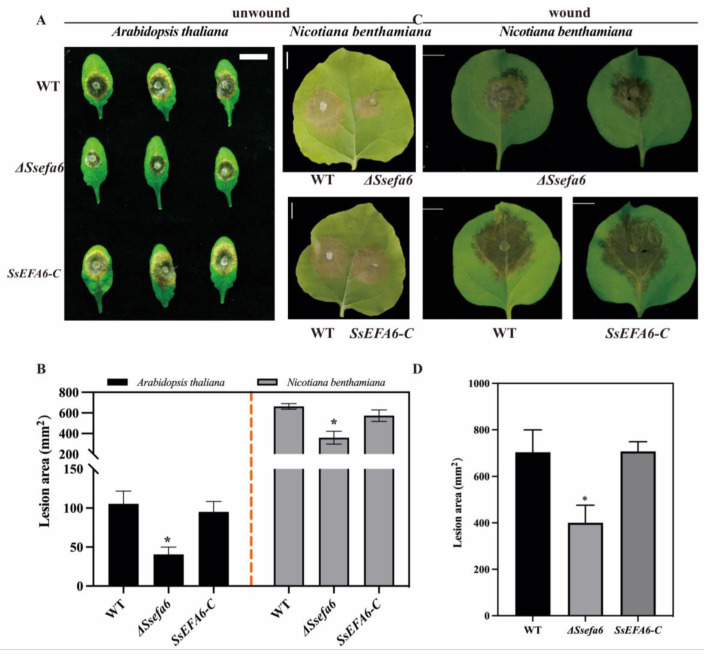
*SsEFA6* participates in the virulence of *S. sclerotiorum*. (**A**,**B**) Infection of *A. thaliana*, and *N. benthamiana* detached leaves by *S. sclerotiorum* and the corresponding infection area. (**C**,**D**) Infection of wounded *N. benthamiana* detached leaves by *S. sclerotiorum* and the respective infection area. Scale bar, 10 mm; the experiment was performed at least three times, yielding consistent results. Error bars denote standard deviation. Statistical analysis comparing WT and *ΔSsefa6* or *SsEFA-C* was carried out using Student’s *t*-test (n = 6, * *p* < 0.05). WT is the wild-type strain, *ΔSsefa6* is the knockout strain, and *SsEFA-C* is the complementary strain.

To determine the role of *SsEFA6* in the pathogenic process, the *ΔSsefa6* and WT strains were inoculated onto intact *A. thaliana* and *N. Benthamian* leaves. As expected, the *ΔSsefa6*, WT, and *SsEFA6-C* strains were able to induce necrosis on *A. thaliana* and *N. Benthamian* leaves after 48 h of infection; however, the necrotic area induced by *ΔSsefa6* was significantly smaller than that induced by WT and *SsEFA6-C* strains (Figure 5A,B). Furthermore, when *ΔSsefa6*, WT, and *SsEFA6-C* strains were inoculated onto *N. Benthamian* leaves with wounds, the pathogenicity of *ΔSsefa6* showed partial recovery after 36 h of infection, although the infection area was still significantly less than that of WT and *SsEFA6-C* strains, indicating that *SsEFA6* is involved in the virulence of *S. sclerotiorum* (Figure 5C,D).

### 3.6. SsEFA6 Is Not Involved in Response to Osmotic Stress and Cell Wall Maintenance

**Figure 6:**
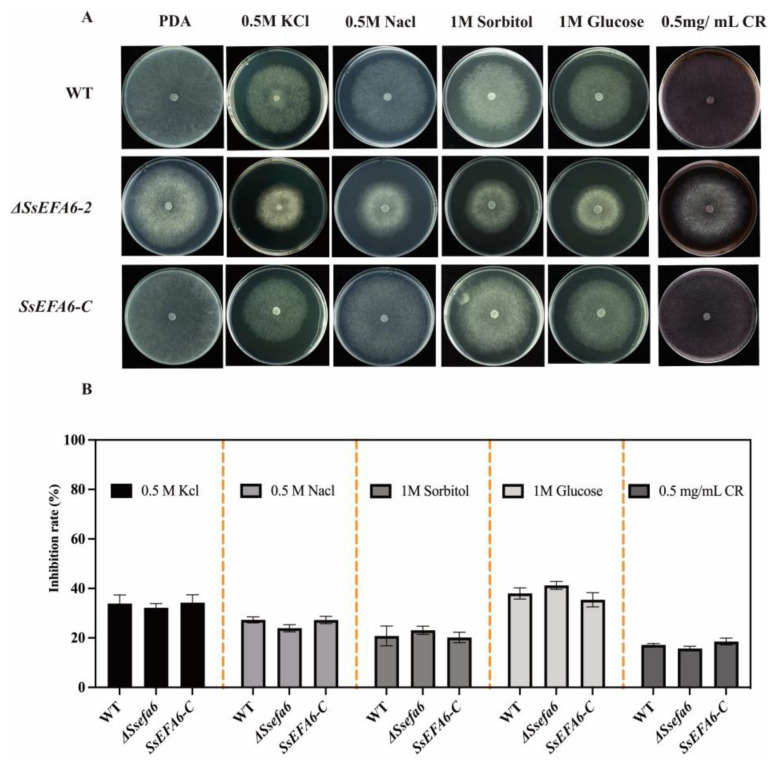
Analysis of abiotic stress response and cell wall integrity of *S*. *sclerotiorum*. (**A**,**B**) The appearance of the colonies and the inhibition rate of WT, *ΔSsefa6*, and *SsEFA6-C* strains were observed on PDA medium with various osmotic stressors or 0.5 mg/mL Congo Red (CR) for 48 h. The experiment was repeated at least three times with consistent results. Error bars show standard deviation. WT is the wild-type strain, *ΔSsefa6* is the knockout strain, and *SsEFA-C* is the complemented strain.

In order to investigate the role of *SsEFA6* in response to osmotic stress in *S. sclerotiorum*, we simulated salt or/and osmotic stress conditions by adding exogenous osmotic stressors (0.5 M NaCl, 0.5 M KCl, 1 M Sorbitol, and 1 M Glucose) to the PDA culture medium. As shown in Figure 6, the *ΔSsefa6* strain displayed no significant difference in response to osmotic stress compared to WT and *SsEFA6-C* strains. Furthermore, the *ΔSsefa6* strain was not affected by the cell wall synthesis inhibitor CR, similar to WT and *SsEFA6-C* strains. These results indicate that *SsEFA6* is not involved in the osmotic stress response process, as well as the synthesis and integrity maintenance of the cell wall in *S. sclerotiorum*.

## 4. Discussion

In this study, reverse genetics was employed to investigate the ADP-ribosylation factor 6 guanine nucleotide exchange factor *SsEFA6* in the *S. sclerotiorum*. Bioinformatic analysis revealed the presence of three homologous proteins to *Ss*EFA6 in the *S. sclerotiorum*, which exhibited high homology to SEC7 domain-containing proteins in *B. cinerea*, *M. fructicola*, and *M. laxa*. However, this conservation was not observed in *F. graminearum*, *F. oxysporum* and *M. oryzae*. This reveals that the evolution of the *Ss*EFA6 protein is diverse in plant pathogenic fungi, suggesting the potential of multiple biological functions.

Deletion of the *FgCDC25* gene in *F. graminearum* results in reduced formation of toxin bodies and decreased production of deoxynivalenol (DON), leading to the loss of the ability to form infection structures and significantly reduced infectivity in plants [42]. Furthermore, another guanine nucleotide exchange factor in *F. graminearum, Fg*Bud3, exhibits similar biological functions. Deletion mutants of *FgBud3* show significant restrictions in fungal nutrient acquisition, development, spore germination, plant infection, and toxin production. This indicates the crucial importance of guanine nucleotide exchange factors in the growth, development, and virulence of plant pathogenic fungi [43]. Our study extended this finding by investigating the guanine nucleotide exchange factor *Ss*EFA6 in *S. sclerotiorum*. The deletion of the guanine nucleotide exchange factor *Ss*EFA6 resulted in significant alterations in the colony morphology of *S. sclerotiorum*. Compared to WT and *SsEFA6-C* strains, the *ΔSsefa6* strain exhibited anomalies in hyphal branching patterns, impaired formation of sclerotia, and a substantial reduction in pathogenicity.

**Figure 7:**
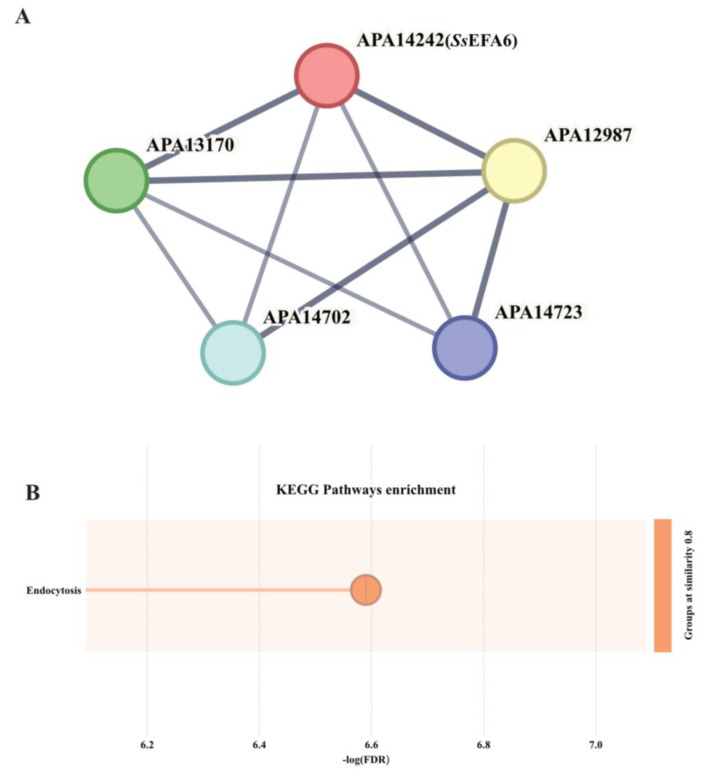
Construction of the protein network for *Ss*EFA6 and KEGG enrichment analysis. (**A**) Interaction network of *Ss*EFA6 protein, where line thickness reflects the strength of data support. Minimum required interaction score = 0.7. (**B**) KEGG enrichment analysis of interacting proteins of *Ss*EFA6, where the size of circles indicates the number of enriched proteins in the pathway. Protein–protein interaction network analysis and KEGG pathway annotation were carried out using the online software STRING (12.0).

Moreover, the guanine nucleotide exchange factors in *F.graminearum* have revealed their potential involvement in biological functions closely related to vesicle transport, endocytosis, and autophagy. For instance, the guanine nucleotide exchange factor *Fg*Mon1 not only participates in processes such as nutrient growth, development, spore germination, morphology, plant infection, and toxin production but also serves as a crucial regulator of vesicle transport, endocytosis, and autophagy [44]. In order to investigate the biological functions of vesicle transport and endocytosis associated with *Ss*EFA6, we utilized STRING to identify proteins that may interact with *Ss*EFA6 and conducted enrichment analysis using KEGG pathways. The protein network for *Ss*EFA6 and KEGG enrichment analysis (Figure 7) suggests that loss of *Ss*EFA6 disrupts the protein interaction network it involves, impacting the endocytic function of the fungus, ultimately leading to weakened virulence, abnormal formation of appressorium and sclerotia. However, these potential discoveries require extensive validation through rigorous experiments in the future.

Compound appressorium and oxalic acid play a crucial role in the interactions between *S. sclerotiorum* and its host. The formation of a compound appressorium to breach the host’s cuticle and cell wall [12]. Oxalic acid, a crucial virulence factor, boosts hydrolytic enzyme activity [45], triggers plant cell death [46], and suppresses host defense mechanisms [47]. Here, the absence of *SsEFA6* led to abnormal development of the compound appressorium but did not affect the secretion of oxalic acid in *S. sclerotiorum*. When we examined the cell wall integrity of the *ΔSsefa6* strain, we found that *SsEFA6* was not essential for *S. sclerotiorum* to maintain cell wall integrity. This suggests that *SsEFA6* might be involved in *S. sclerotiorum*’s interactions with its host via mechanisms beyond the oxalic acid pathway.

## 5. Conclusions

In summary, our findings demonstrate the involvement of *SsEFA6* in mycelial development, as well as sclerotial and compound appressorium formation in *S. sclerotiorum*, thereby contributing to the infection process and virulence of the fungus towards host plants, providing evidence for a better understanding of the role of *SsEFA6* in *S. sclerotiorum*.

## Data Availability

Protein files of *B*. *cinerea* (version Botrytis_cinerea.ASM83294v1), *F*. *graminearum* (version Fusarium_graminearum.RR1), *F*. *oxysporum* (version Fusarium_oxysporum.FO2), *M*. *oryzae* (version Magnaporthe_oryzae.MG8), *M*. *fructicola* (version Monilinia_fructicola_gca_008692225.ASM869222v1), and *S*. *sclerotiorum* (version Sclerotinia_sclerotiorum_1980_uf_70_gca_001857865.ASM185786v1) were obtained from the EnsemblFungi database (https://fungi.ensembl.org/).

## Supplementary Materials

The following supporting information can be downloaded at https://www.mdpi.com/article/10.3390/jof11110821/s1, Table S1: Amplification and detection primers for *SsEFA6*; Figure S1: Infection assessment and mutation site analysis of mutant strain 2WX2-11; Figure S2: Homologous recombination-based split-marker was employed to knock out *SsEFA6*; Figure S3: Screening of homozygous transformants of *∆Ssefa6* based on protoplast transformation; Figure S4: Selection and PCR detection of the *SsEFA6-C* transformants based on protoplasts transformation.
