# Supplementary material for: The Guanine Exchange Factor SsEFA6 Participates in Appressorium Formation and Virulence in Sclerotinia sclerotiorum
Source: J Fungi (Basel). 2025 Nov 20;11(11):821. doi: 10.3390/jof11110821 (PMC12653896; doi:10.3390/jof11110821)
Supplement: Supplementary file 1 [file jof-11-00821-s001.zip › jof-3894486-supplementary.pdf]

Journal of
*Fungi*

MDPI

*Article*

# Supplementary: The guanine exchange factor *SsEFA6* participates in appressorium formation and virulence in *Sclerotinia sclerotiorum*

**Kunmei Wang†, Ting Wang†, Qi Xia, Na Xie, Jiancheng Cao and Shitou Xia ***

Hunan Provincial Key Laboratory of Phytohormones and Growth Development, College of Bioscience and Biotechnology, Hunan Agricultural University, Changsha 410128, China; yuhunan@stu.hunau.edu.cn (K.W.); tina@stu.hunau.edu.cn (T.W.); xiaqi@stu.hunau.edu.cn (Q.X.); NaXie@stu.hunau.edu.cn (N.X.); caojiancheng@stu.hunau.edu.cn

* Correspondence: xstone0505@hunau.edu.cn.
† These authors contributed equally to this work.

Academic Editor: Firstname Lastname

Received: date
Revised: date
Accepted: date
Published: date

**Citation:** To be added by editorial staff during production.

**Copyright:** © 2025 by the authors. Submitted for possible open access publication under the terms and conditions of the Creative Commons Attribution (CC BY) license (https://creativecommons.org/licenses/by/4.0/).

Journal of Fungi

**Supplementary Table S1.** Amplification and detection primers for *SsEFA6*. 13

| **Primer Name** | **sequence** |
|---|---|
| 1F | CTCAACCAATCAATCTACCATC |
| 2R | GTGCTCCTTCAATATCATCTTCTGTGAGTCGTCATGAAGTATTGTAG |
| 3F | AGGTACACTTGTTTAGAGGTAATCCATACGACACGGATGACTC |
| 4R | CCAAGGTGATCCAGCATA |
| HYGF | ACAGAAGATGATATTGAAGGAGCAC |
| HYGR | GGATTACCTCTAAACAAGTGTACCT |
| YGF | TCTCGGAGGGCGAAGAATCTCGTGC |
| HYR | GCATCATCGAAATTGCCGTCAACC |
| SsTubqF | ACCTCCATCCAAGAACTC |
| SsTubqR | GAACTCCATCTCGTCCAT |
| *SsEFA6*_checkF | CGTGGACCTGAATGTTGA |
| *SsEFA6*_checkR | ACCTCTGATTGCTTGTAACT |
| Neomycin_checkF | CAGAAGATGATATTGAAGGAGCAC |
| Neomycin_checkR | GGATTACCTCTAAACAAGTGTACCT |
| SeqF_360 | CGGCATACCTGGATTAGGAC |
| SeqR_360 | CTGACTTTGGCATCTTCTTGAG |
| SeqF_120 | GTTGCTCCACTTTCCATTTG |
| SeqR_120 | GCGTCAAGATCCTGATAACC |
| SeqF_780 | ACCTTAACTCTCGTCCACT |
| SeqR_780 | CCATTCTGAGTCCGTAGC |

A

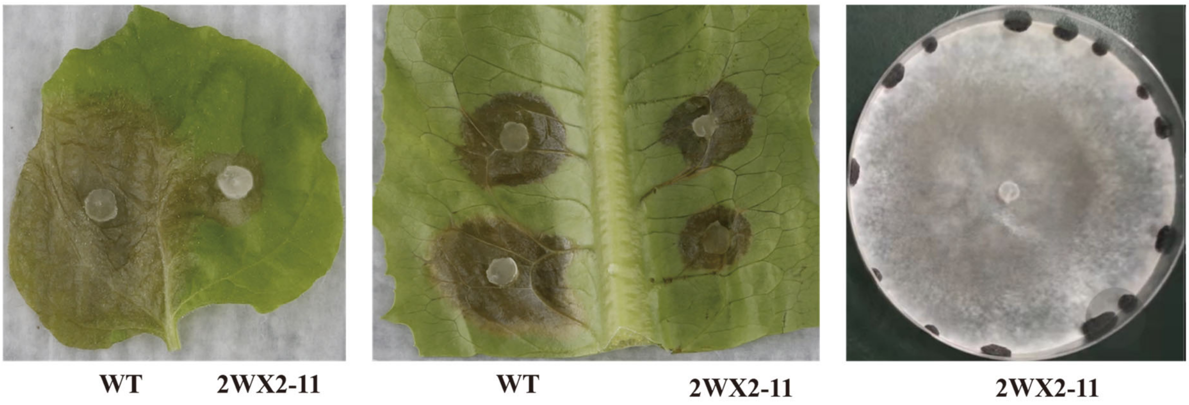


B

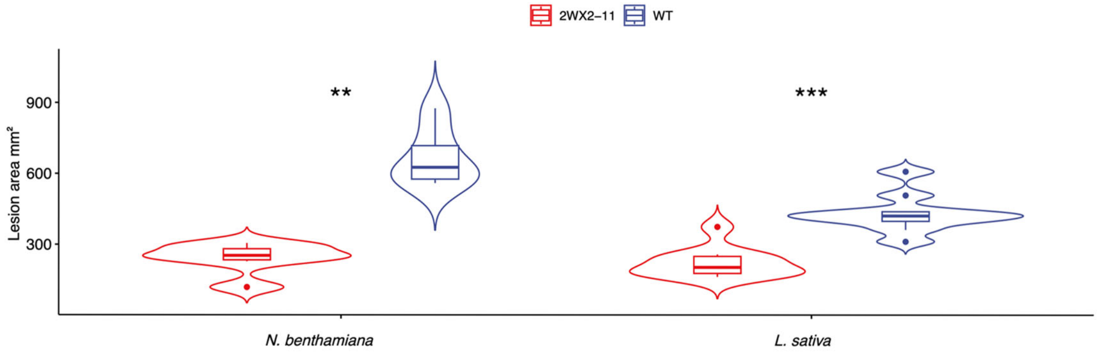


C

| Chromosome | Nucleotide change | AA change | Reference gene | Annotation |
|---|---|---|---|---|
| 1 | C200T | S67L | sscle_01g009360 | H/ACA ribonucleoprotein complex non-core subunit NAF1 |
| 4 | G1762T | D588Y | sscle_04g033780 | Flavoprotein domain-containing protein |
| 12 | C3358T | S1104F | sscle_12g090120 | SEC7 domain-containing protein |

D

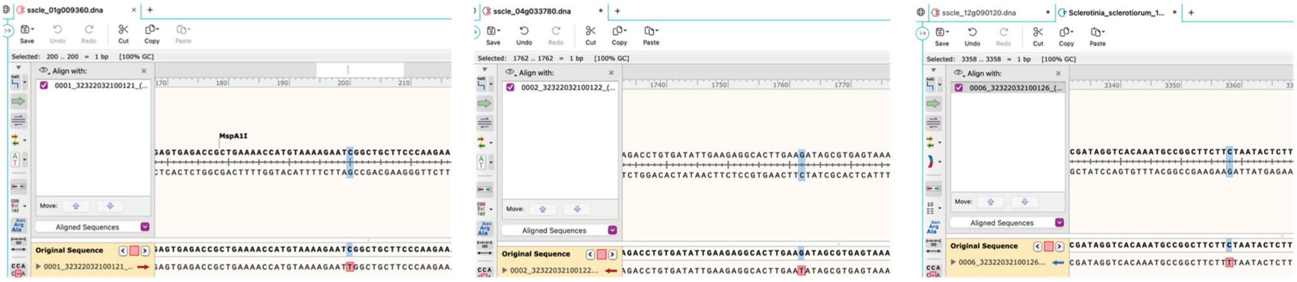

**Supplementary Figure S1.** Assessment of infectivity and mutation site detection of mutant strain 2WX2-11. (A) Infection of N. benthamiana and Lactuca sativa var. asparagina L.H.Bailey ex Holub leaves by 2WX2-11. (B)Infection of N. benthamiana L. sativa detached leaves by S. sclerotiorum and the corresponding infection area. (C) Candidate genes associated with reduced infectivity of mutant strain 2WX2-11. (D) Detection of mutation sites in candidate genes by next-generation sequencing. The experiment was performed at least three times, yielding consistent results. Statistical analysis

comparing the wild-type (WT) and 2WX2-11 strains was carried out using Student's t-test (*p<0.05).

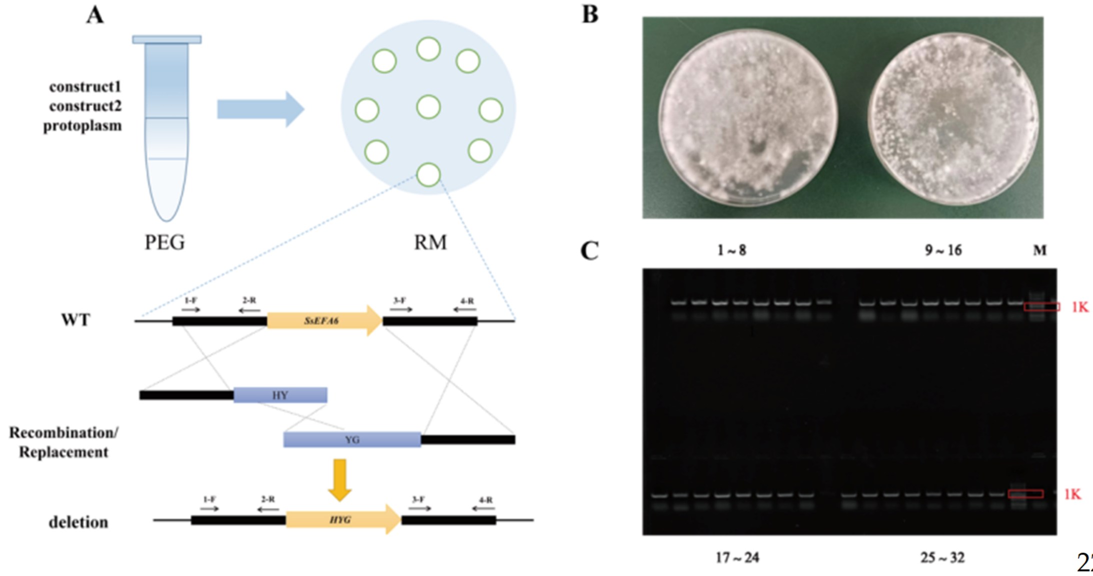


**Supplementary Figure S2.** Homologous recombination-based split-marker was employed to knock out *SsEFA6*. **(A)** Schematic diagram illustrating the knockout of *SsEFA6* using the split-marker method. **(B)** Phenotypic analysis of positive protoplast transformants. **(C)** PCR detection of *SsEFA6*-UP-HY (lane1-16) and *SsEFA6*-Down-YG (lane17-32) in the transformants.

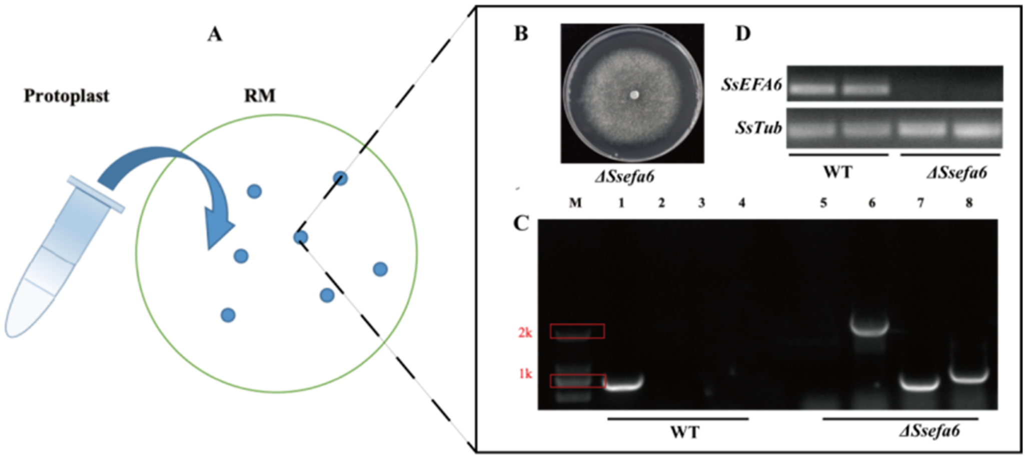


**Supplementary Figure S3.** Screening of homozygous transformants of *ΔSsefa6* based on protoplast transformation. **(A)** Schematic diagram of the screening of homozygous transformants of *ΔSsefa6*. **(B)** Phenotypic analysis of positive protoplast transformants. **(C)** PCR detection of *SsEFA6* (lane 1, 5), *Hygromycin* gene (lane 2, 6), *SsEFA6*-UP-HY (lane 3, 7) and *SsEFA6*-Down-YG (lane 4, 8) sequence in the transformants. **(D)** Analysis of the expression levels of *SsEFA6* in the *ΔSsefa6* transformants.

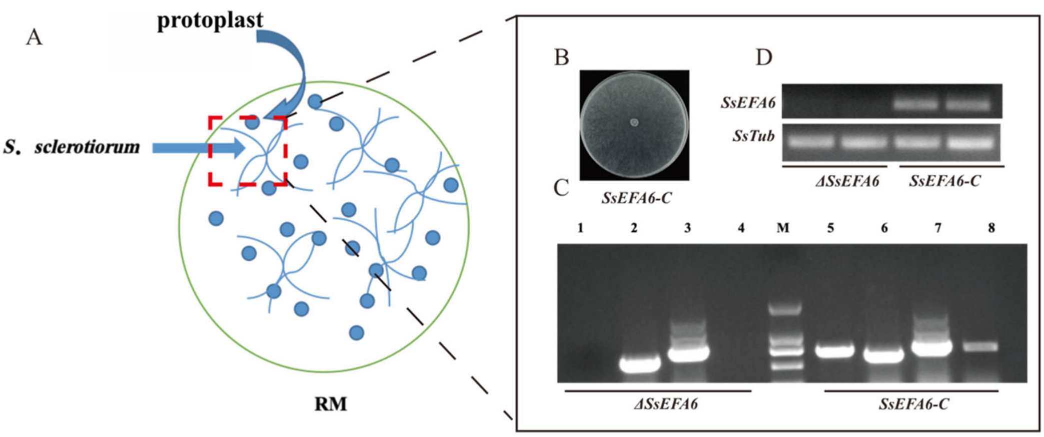


**Supplementary Figure S4.** Selection and PCR detection of the *SsEFA6-C* transformants based on protoplast transformation. **(A)** Schematic diagram of the screening of the *Ss*EFA6-C transformant. **(B)** Phenotypic representation of positive *Ss*EFA6-C transformants. **(C)** PCR detection of *SsEFA6* (lane 1, 5), *SsEFA6*-UP-HY (lane 2, 6), *SsEFA6*-Down-YG (lane 3, 7), and Neomycin gene (lane 4, 8) sequences in the *SsEFA6-C* transformant. **(D)** Analysis of *SsEFA6* expression levels in the *SsEFA6-C* transformants.
